# Comparative Genomic Analysis of Stenotrophomonas maltophilia Strain W18 Reveals Its Adaptative Genomic Features for Degrading Polycyclic Aromatic Hydrocarbons

**DOI:** 10.1128/Spectrum.01420-21

**Published:** 2021-11-24

**Authors:** Yaqian Xiao, Ruhan Jiang, Xiaoxiong Wu, Qi Zhong, Yi Li, Hongqi Wang

**Affiliations:** a College of Water Sciences, Beijing Normal University, Beijing, China; b Key Laboratory of Ecology of Rare and Endangered Species and Environmental Protection (Guangxi Normal University), Ministry of Education, China, Guangxi Normal University, Guilin, Guangxi; c College of Environment and Resources, Guangxi Normal University, Guilin, Guangxi; University of Minnesota

**Keywords:** PAHs, comparative genomic analysis, pangenome, *Stenotrophomonas maltophilia*

## Abstract

Polycyclic aromatic hydrocarbons (PAHs) are hazardous pollutants that are ubiquitous in the environment. Numerous bacteria have evolved to have degrading genes or pathways to degrade PAHs. Stenotrophomonas maltophilia strain W18 was found to be able to degrade PAHs. Including 43 other complete genome sequences of S. maltophilia strains, we performed a comparative genomic analysis of 44 S. maltophilia strains by running OrthoFinder. A KEGG pathway enrichment analysis of environmental and clinical isolates of S. maltophilia revealed that environmental isolates tended to enhance gene functions such as “energy metabolism,” “amino acid metabolism,” “xenobiotic biodegradation and metabolism,” and “folding, sorting, and degradation.” The pangenome of the 44 S. maltophilia strains was open, while the core genome was estimated to reach a steady plateau. Based on gene annotations, we inferred that most of the degradation potential came from the core genome of S. maltophilia, while character genes and accessory genes also contributed to the degradation ability of S. maltophilia W18. The genes expression level of core genes, character genes and accessory genes were proved by RT-qPCR experiment, and accessory genes encoding alcohol dehydrogenase were upregulated most compared with genes with similar functions. We performed a credible comparative genomic analysis of S. maltophilia strains. S. maltophilia W18 was set as a model PAH-degrading bacterium of this species in this study, which would provide guidance for understanding and predicting the degradation mechanisms of other PAH-degrading S. maltophilia strains lacking complete genome data or waiting to be determined.

**IMPORTANCE** This study provided the latest comparative genomic analysis on Stenotrophomonas maltophilia strains and focused on analyzing their genomic features that allow them to adapt to natural environments. In this study, we set S. maltophilia W18 as a typical PAH-degrading strain of this species. By discussing the genomic adaptative features of degrading PAH, we can predict genomic adaptative features of other S. maltophilia PAH-degrading strains since the core function of this species is stable. The gene functions of how S. maltophilia environmental isolates are enhanced for adaptation to various natural environments compared with clinical isolates have been revealed. Combined with a pangenome analysis and RT-qPCR results, we have proved that core genes, character genes, and accessory genes are all involved in PAH degradation. Accessory genes encoding alcohol dehydrogenase were upregulated most compared with core and character genes with similar functions, which suggests that PAH metabolization potential might be enhanced by horizontal gene transfer.

## INTRODUCTION

Polycyclic aromatic hydrocarbons (PAHs) are one of the most ubiquitous classes of organic compounds; are commonly detected in the environment; and are believed to be genotoxic, mutagenic, and carcinogenic (1, 2). Bioremediation is considered an economical and high-efficiency method to eliminate PAH pollution (3). Many studies have focused on studying diverse PAH-degrading bacteria, such as members of the genera Pseudomonas, Mycobacterium, *Rhodococcus*, *Stenotrophomonas*, *Burkholderia*, and *Sphingomonas* (4–8). Studies have determined that PAH-degrading bacteria possess PAH degradation genes to catabolize aromatic ring cleavage of initial PAHs and aromatic intermediates (9, 10); they have mostly studied aromatic hydroxylation dioxygenase, which initiates the dioxygenation of PAHs (10, 11). Some studies also refer to PAHs degradation genes as genes that play a role during PAH degradation, including genes that could maintain cellular homeostasis, detoxify reactive metabolites, and interact with PAHs (5, 12, 13). Bacteria isolated from the natural environment that evolved to possess PAH degradation genes could also have evolved to adapt to their habitats (9, 14, 15).

Performing a comparative genomic analysis based on genome data of available strains could reveal the common species characteristics of large populations and reveal the physiological characteristics of special strains. Many PAH degradation-associated genes of Mycobacterium strains were shared among species, and some PAH degradation-associated genes were obtained by horizontal gene transfer (9). Stenotrophomonas maltophilia environmental isolates have been shown to carry as many multidrug resistant efflux pumps as clinical isolates, and they lacked efflux pumps possessed by clinical isolates (16). Previous comparative genomic studies of S. maltophilia strains focused mostly on their pathogenicity, while fewer comparative genomic studies have examined environmental isolates and discussed their bioremediation potential (16–18).

S. maltophilia strain W18 was isolated from crude oil-contaminated soil and showed high efficiency at degrading fluoranthene, a kind of PAH (19). S. maltophilia W18 could degrade 60% fluoranthene in culture in 10 days (19). In addition, S. maltophilia strains AJH1, C6, and SVIA2, among others, were also determined to possess a PAH degradation ability (7, 20, 21). Although many S. maltophilia strains possess a great PAH degradation ability, limited complete genome data of S. maltophilia have been deposited in public databases. We need a series of analysis methods to understand and conclude the origin and constitution of their degradation potentials and to perform a comparative genomic analysis. To ensure the accuracy of the comparative genomic analysis, we chose only complete genome data deposited in the NCBI database (44 S. maltophilia genomes, which include S. maltophilia W18). Reverse-transcription quantitative PCR (RT-qPCR) is a common approach to measure the expression of genes. Combined with a comparative genomic analysis and gene function results proven by RT-qPCR experiments, we preliminarily described the mechanisms by which S. maltophilia adapts to natural environments and how S. maltophilia W18 evolves to possess a PAH degradation potential, thereby better understanding the species characteristics and degradation mechanisms of S. maltophilia.

## RESULTS

### Pangenome analysis.

**FIG 1:**
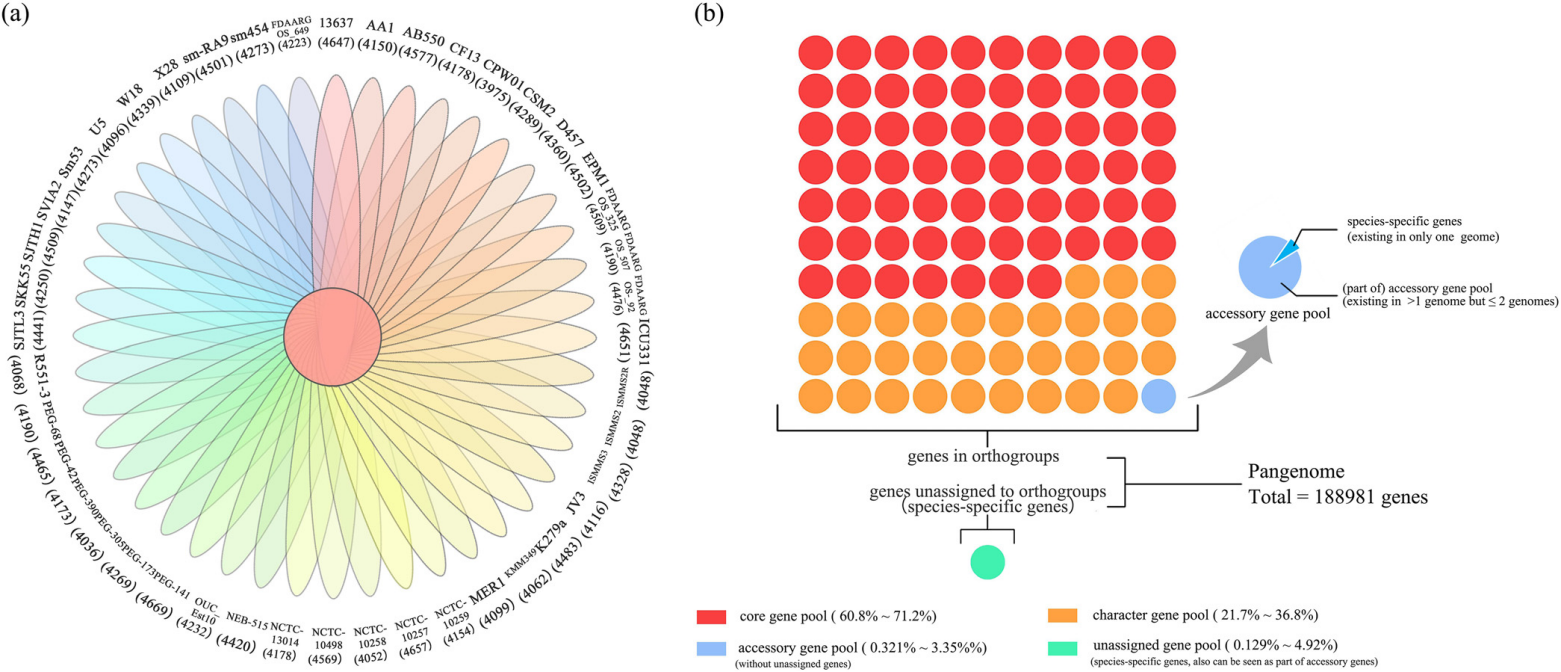
Overall orthogroup statistics. (a) Comparison of orthogroups by OrthoFinder. The outermost layer indicates the names of the 44 S. maltophilia strains, and the numbers in the parentheses are the numbers of coding sequences (CDSs) of each strain. The numbers in black in the Venn diagram are the numbers of orthogroup-containing species. Under those statistics, numbers highlighted in red in the Venn diagram reflect the CDS numbers of each strain in the core genome. (b) Classification and percentage of each gene pool constituting the pangenome of the 44 S. maltophilia strains.

**FIG 2:**
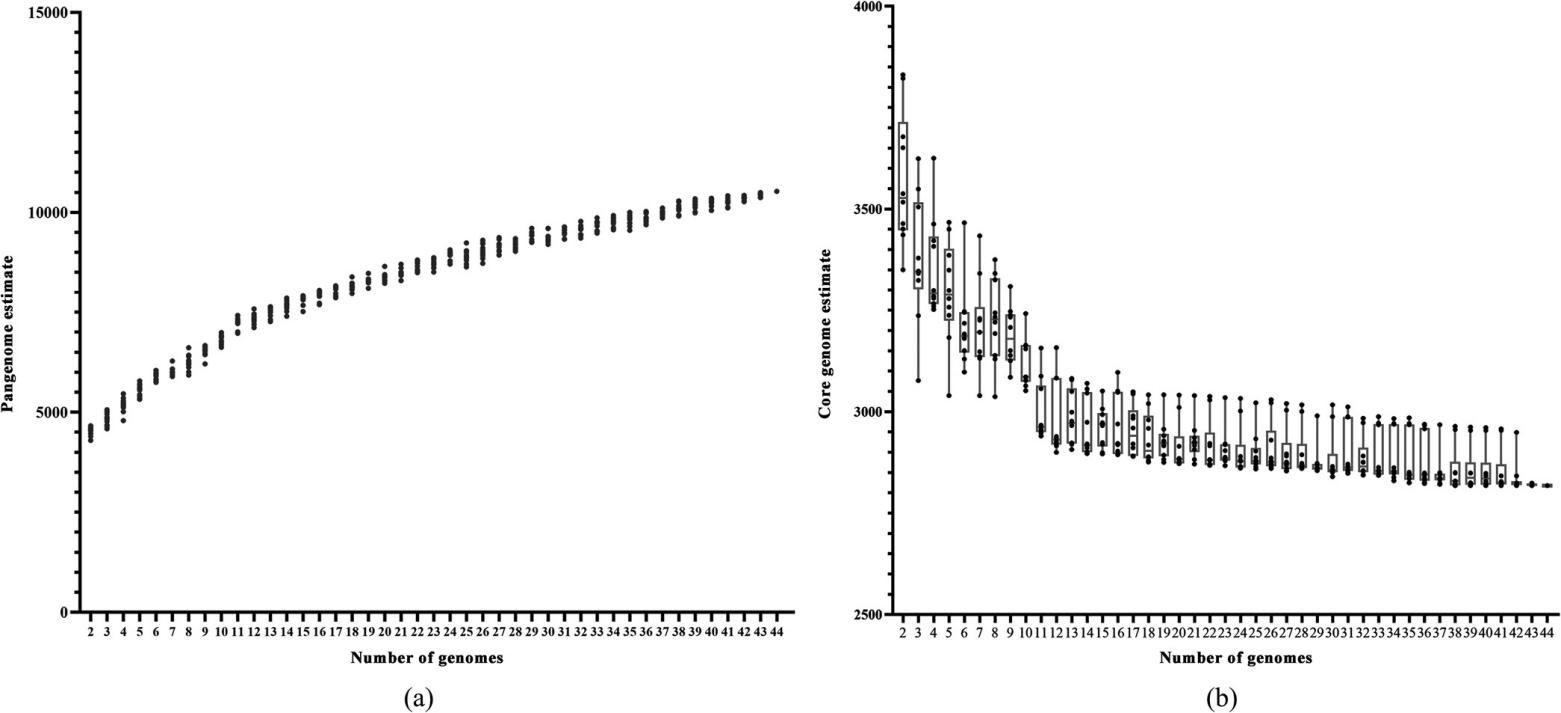
Pangenome and core genome estimate. (a) Pangenome estimate. The *x* axis shows the number of genomes, and the *y* axis shows the pangenome size (number of genes). (b) Core genome estimate. The *x* axis shows the number of genomes, and the *y* axis shows the pangenome size (number of genes) of S. maltophilia strains. The pangenome and strict core genome estimates are expressed as the number of orthogroups plotted against the number of genomes. Each point represents the pangenome or the strict core genome estimate of one combination.

According to the calculation results exported by OrthoFinder, there were 188,981 genes in total among the 44 strains, which could also be referred to as the pangenome of the 44 S. maltophilia strains. On average, 66.2% of the genes of each genome constituted the core genome, which is reflected in the middle of Fig. 1a. In the S. maltophilia W18 genome, 2,818 orthogroups existing in the 44 genomes constituted the core gene pool (core genome), 1,105 orthogroups in less than 2 genomes were classified into the accessory gene pool (accessory genome), and 3,650 orthogroups existing in less than 44 genomes but more than 2 genomes were referred to as the character gene pool (character genome). We noticed that orthogroups present only in one genome could also be seen as species-specific orthogroups. Both species-specific genes and unassigned genes present only in one genome could also be referred to as accessory genes. The percentage of the pangenome and genes in each gene pool is shown in Fig. 1b. As reflected in Fig. 2, the number of core genomes decreased gradually, and the pangenome increased gradually with the increase in the number of given genomes. The function of the fitting curve was “*n* = 3549.479*N^−0.287^.” The α value was 0.287 (α < 1), indicating an open pangenome. Compared with the pangenome, the size of the core genome gradually reached a steady-state approximation.

### Phylogenetic tree analysis.

**FIG 3:**
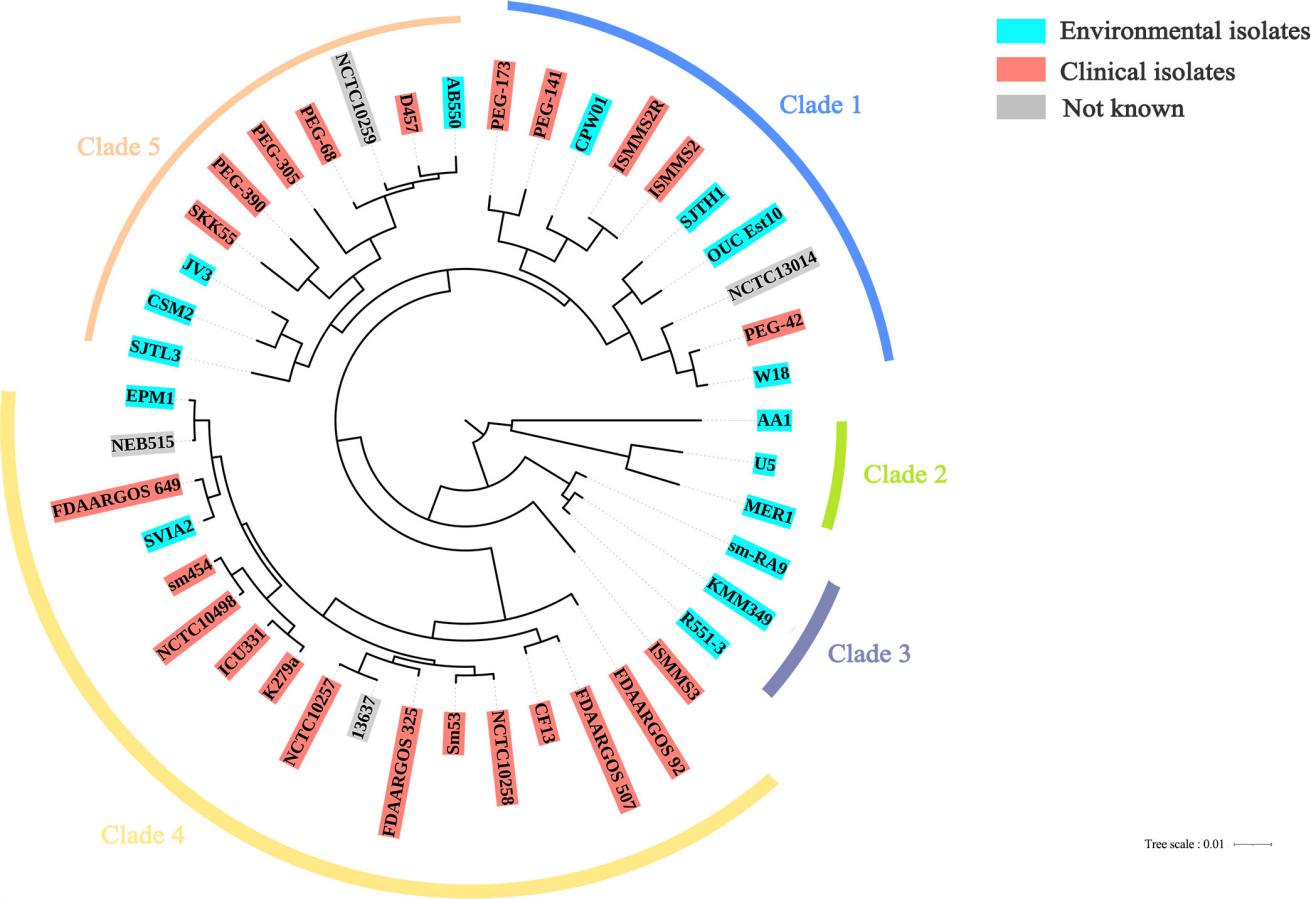
Phylogenetic tree of the 44 S. maltophilia strains.

As shown in Fig. 3, in the phylogenetic tree, the 44 strains were clustered into 5 clades, and the habitat information for each strain was mapped onto the tree. Seventeen strains, of which most were isolated from human clinical samples, constituted the largest clade in the tree, clade 4. Clade 2 and clade 3 were characterized by strains isolated only from the environment. Clades 1 and 5 contained strains isolated from humans and the environment. The PAH-degrading strain S. maltophilia W18 was included in clade 1. The 44 S. maltophilia strains were not distributed strictly according to their isolation sites in the phylogenetic tree.

### Gene category enrichment analysis.

**FIG 4:**
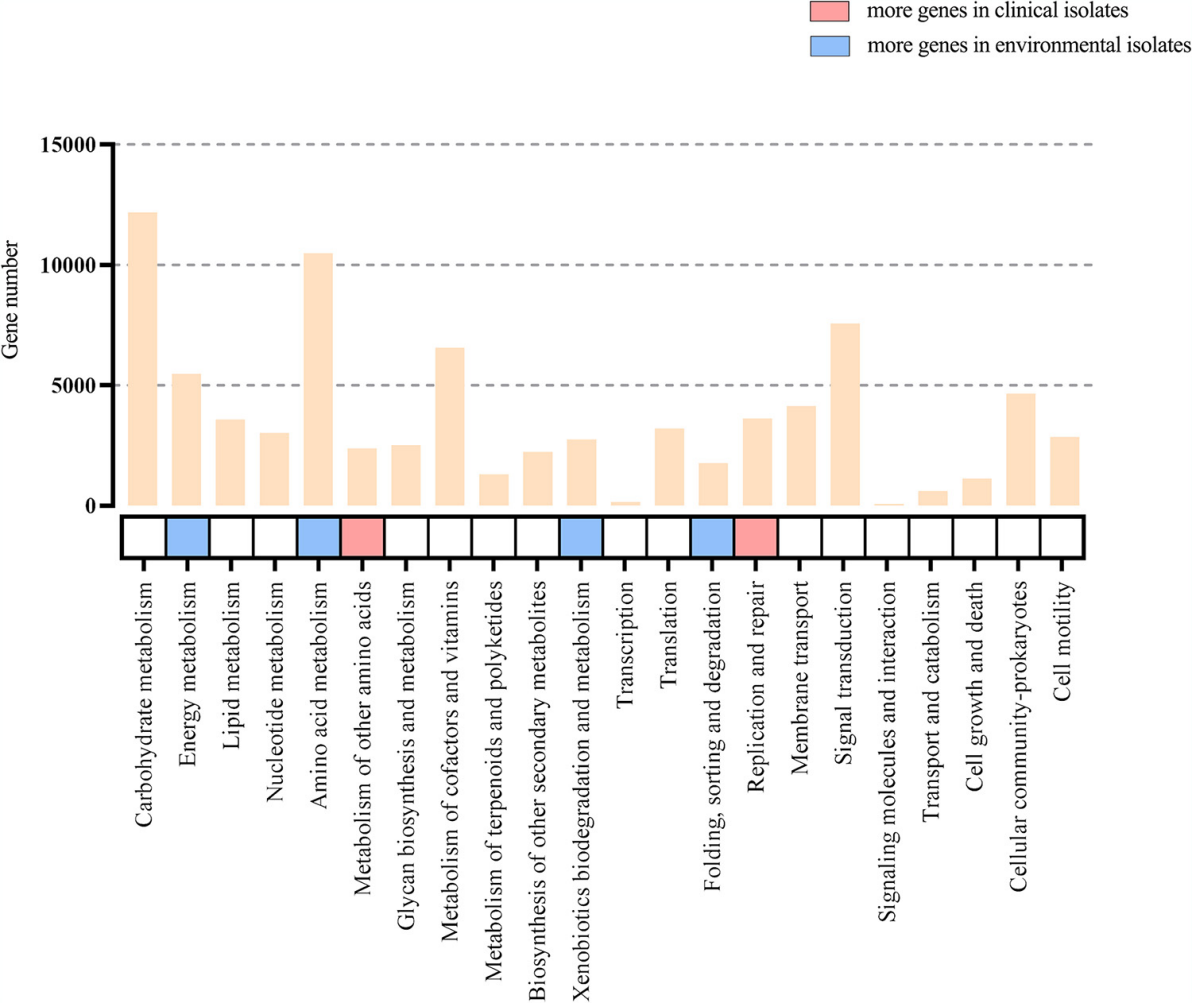
KEGG pathway comparison between environmental and clinical S. maltophilia isolates. The *x* axis indicates KEGG functional categories, and the bar charts represent the total number of counted genes for each category of KEGG annotation. The enrichment or depletion of each gene category based on *t* test results is shown on the heatmap. Blank cells indicate that there was no significant difference between the genomes from the two kinds of habitats. Colored cells indicate significant differences between strains isolated from different sources (*P* < 0.05). The legend shows the mean ratio of the annotation number between environmental isolates and clinical isolates in the corresponding category.

Hypergeometric tests showed that most of the KEGG secondary pathways were composed of core genes and character genes (see Table S3 in the supplemental material). As shown in Fig. 4, the enrichment analysis using a *t* test showed that a large number of S. maltophilia strains isolated from environmental sources were enriched in 4 KEGG functional categories, as follows: “energy metabolism,” “amino acid metabolism,” “xenobiotics biodegradation and metabolism,” and “folding, sorting and degradation.” On the other hand, clinical isolates of S. maltophilia were more highly enriched in the categories “metabolism of other amino acids” and “replication and repair.” The KEGG category *t* test results for environmental isolates and clinical isolates are shown in Table S3.

### KEGG annotation.

**FIG 5:**
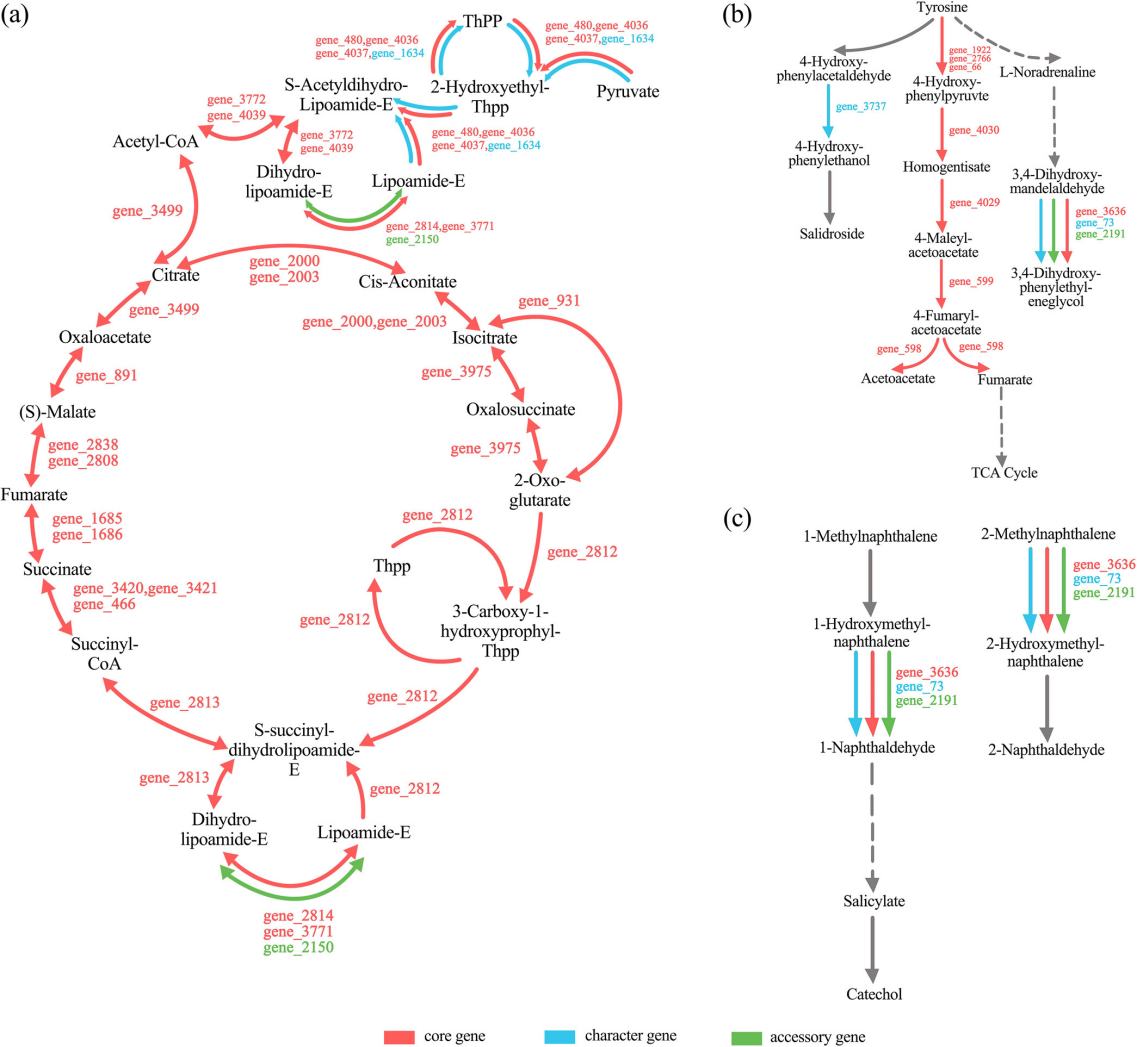
Part of the “TCA cycle,” “tyrosine metabolism,” and “naphthalene degradation” pathways. (a) TCA cycle (not complete). (b) Tyrosine metabolism pathway (not complete). (c) Naphthalene degradation pathway (not complete). Gene numbers of core, character, and accessory genes are shown in the corresponding colors. Dashed arrows indicate two or more successive reactions, whereas solid arrows indicate successive reactions. Colored arrows indicate that genes classified into the corresponding category were annotated, and gray arrows indicate that no gene taking part in the conversion was annotated.

According to the KEGG and eggNOG annotation results, we predicted 59 possible PAH degradation genes of W18 based on their gene function (see Table S8 in the supplemental material). Annotations showed that 27 genes in the whole genome of S. maltophilia W18 constituted the complete tricarboxylic acid (TCA) cycle, of which 25 genes belonged to the core gene pool, gene_2150 came from the accessory gene pool, and gene_1634 came from the character gene pool. KEGG pathway annotations showed that core genes could normally constitute the complete physiological pathway, as shown in Fig. 5a. As shown Fig. 5b, tyrosine could also be metabolized through core genes. Moreover, some character genes and accessory genes have similar functions to core genes. Similar to core gene_480, gene_4036, and gene_4037, character gene_1634 could also take part in the conversion of pyruvate to S-acetyldihydrolipoamide-E. In the naphthalene degradation pathway, core gene_3636, character gene_73, and accessory gene_2191 all encode alcohol dehydrogenase, as is reflected by Fig. 5c. Genes in the “TCA cycle,” “tyrosine metabolism” and “naphthalene degradation” pathways are shown in Table S4, S5, and S6.

### Real-time quantitative PCR.

**FIG 6:**
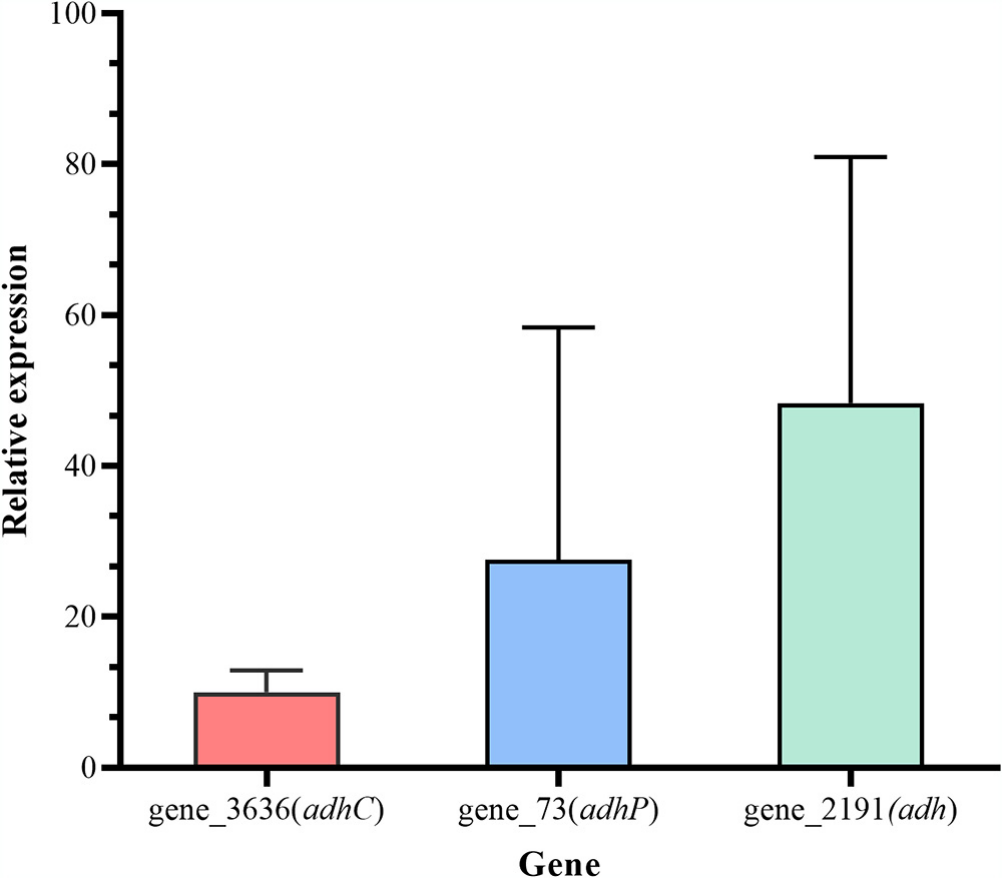
Relative expression of *adhC*, *adhP*, and *adh* in S. maltophilia W18 incubated in 20 mg/liter FLA-MSM.

The core gene_3636 (*adhC*), character gene_73 (*adhP*), and accessory gene_2191 (*adh*) were all predicted to encode the same product. Therefore, we analyzed the relative expression of these three genes between S. maltophilia W18 incubated in fluoranthene-mineral salt medium (FLA-MSM) and Glu-MSM after 24 hours (Fig. 6). The fact that all relative expression ratios of these three genes increased reflected the importance of alcohol dehydrogenase when S. maltophilia W18 degraded fluoranthene (Fig. 6). Meanwhile, accessory gene_2191 was upregulated most among the three genes, indicating that it may play a more important role during PAH degradation than core and character genes with similar functions.

## DISCUSSION

The 44 S. maltophilia strains exhibited an open genome pattern, and numerous new genes were observed when each new genome was added to the examined genomes. The open pangenome shows great potential for use in the discovery of novel genes as more S. maltophilia strains are sequenced and provides bacteria with great potential to adapt to new niches (22, 23). The core genome estimation of the 44 S. maltophilia strains showed a declining trend, indicating an increase in diversity among organisms and stable core genome function (24). In addition to PAH degradation potential, former studies on some S. maltophilia strains have revealed their bioremediation potential for heavy metal accumulation or estrogen degradation (25, 26). As each branching clade was not strictly formed by clinical or environmental isolates, S. maltophilia strains presented a free-living style (27). Combined with pangenome analysis results, S. maltophilia could be considered a promising bioremediation species that could cope with a highly complex polluted environment and be conveniently applied to *in situ* bioremediation.

KEGG pathway enrichment analyses have shown differences between clinical isolates and environmental isolates of S. maltophilia strains. KEGG annotation indicated that the environmental isolates tended to adapt to the environment by enhancing functions such as “xenobiotics biodegradation and metabolism,” “folding, sorting and degradation,” “amino acid metabolism,” and “energy metabolism.” In the natural environment, bacteria need to utilize diverse and complex compounds as carbon sources, such as PAHs, environmental estrogens, and trichloroethylene (7, 26, 28). Xenobiotics, including PAHs, are defined as chemical compounds that are persistent in the environment and foreign to organisms and have hazardous effects on ecosystems (29). Organisms living in a natural environment exposed to xenobiotic compounds utilize them as essential energy sources, so the bioavailability of various such substances can be determined (11, 20, 29). Xenobiotic compounds, such as PAHs, can cause gene mutations (1). Therefore, folding, sorting, and degradation of proteins are important mechanisms to maintain cellular and organismal homeostasis by removing faulty proteins (30). The processes of amino acid metabolism have been proven to be vital for coping with environmental stress, as well as adapting to the natural environment (31, 32). Pathogens can use amino acids, fatty acids, and lipids, which are abundant and readily assimilated in the human body, as energy sources, leading to a tendency toward a reduction in energy metabolism (33). Compared with the S. maltophilia environmental isolates, the clinical isolates were significantly enriched with genes classified into “metabolism of other amino acids” and “replication and repair.” According to the KEGG pathway annotation, some genes annotated to “metabolism of other amino acids” are also involved in antibiotic resistance, such as vancomycin resistance, which provides a survival advantage to bacteria in the human body. The processes of DNA replication and repair were found to be vital for pathogens to effectively colonize the human host (34, 35). Many studies have investigated key genes or factors involved in bacterial degradation of PAHs using omics research methods (36–38). Our study also offered a systematic method for filtering important gene categories by using comparative genomic methods. Since genomic adaptative mechanisms have evolved in the natural environment, this method might be more straightforward and closer to the real situation.

The diversity of genes present in different pools indicates that strains use different strategies to adapt to various environments (39). The core set of genes is under high selective pressure for functions that prevent drastic changes (40). Alpha/beta hydrolases can catalyze carbon-carbon bonds of various aromatic compound degradation intermediates (41). Both core gene_3224 and core gene_4285 encode proteins that belong to the alpha/beta hydrolase family. PAH oxidation can produce a number of reactive oxygen species that are normally produced within cells and that are toxic to biological systems (38). Nine core genes of S. maltophilia W18 (gene_896, gene_1501, gene_1778, gene_2823, gene_2835, gene_3398, gene_3802, gene_3987, and gene_4320) all encode glutathione *S*-transferase, which can detoxify reactive metabolites produced during the process of PAH degradation and function in the metabolic pathway of PAHs (13). Thus, it can be inferred that part of the potential to cope with stimulation by PAHs comes from the basic physiological function of S. maltophilia W18.

Alcohol dehydrogenases are responsible for a series of reactions that convert ring cleavage metabolites to aromatic intermediates before they enter the TCA cycle (38). The TCA cycle is responsible for the lower metabolic pathway of PAHs before the metabolites are completely metabolized (6, 42). The homogentisate metabolism pathway, which is part of the tyrosine metabolism pathway, was predicted to exist in the genomes of all PAH-degrading bacteria (4, 10). KEGG annotations showed that the TCA cycle and homogentisate metabolism pathway could be completed only by core genes. However, gene_2150 was classified as an accessory gene and predicted to encode dihydrolipoamide dehydrogenase, which participates in the TCA cycle. Real-time quantitative PCR analysis results showed that gene_73 (character gene), gene_2191 (accessory gene), and gene_3636 (core gene), which were all predicted to encode alcohol dehydrogenase, were all involved in PAH degradation. The accessory gene_2191 was upregulated the most among these three genes, suggesting the vital role of accessory genes in degraded PAH. Although core genes could normally constitute the complete metabolic pathway, character genes and accessory genes could help to expand the metabolic potential. Since accessory genes possibly originated from horizontal gene transfer (40, 43), part of the PAH degradation potential of S. maltophilia W18 came from horizontal gene transfer.

In conclusion, S. maltophilia is a widely distributed species with increasing application potential in the metabolism or adsorption of pollutants. This study provided a more accurate and solid comparative analysis of S. maltophilia strains and partially revealed the genomic functional characteristics of strains isolated from the natural environment. Since S. maltophilia strains were proven to have an open pangenome pattern, we can therefore predict adaptative mechanisms of other S. maltophilia strains with PAH degradation potential, which might be identified in future studies by thoroughly analyzing S. maltophilia W18. The closed core genome of S. maltophilia strains suggested that most bioremediation potential could be shared among species. However, our research also proved that unique functional genes might be acquired from the natural environment and therefore enhance their bioremediation potential.

## MATERIALS AND METHODS

### Genome sequences and gene prediction.

Genomic DNA of S. maltophilia W18 was extracted, followed by purification with the DNeasy blood and tissue kit (cat no. 69054). Whole-genome sequencing was carried out on the Illumina paired-end 150 (PE150) and PacBio PBRSII platforms. Since the genome of S. maltophilia strain NCTC-10498 had been sequenced twice, the higher-quality assembly was chosen for the following analysis (27). Based on the genome entry deposited before 18 May 2020, 44 complete genome sequences were downloaded before they were analyzed by GeneMarkS (https://topaz.gatech.edu/GeneMark/). Twenty-three strains isolated from human tissue were classified as clinical isolates, while 17 strains isolated from the natural environment were classified as environmental isolates. Four S. maltophilia strains were isolates of unknown origin. Details of the 44 examined strains are shown in Table S1 in the supplemental material.

### Pangenome analysis of the 44 S. maltophilia strains.

Pascal et al. described the pangenome as consisting of the core genome, character genome, and accessory genome (40). Nucleotide sequences and amino acid sequences predicted by GeneMarkS were used for the following pangenome analysis by OrthoFinder (version 2.3.12) (44). The classification method described by Pascal et al.(40) distinguishes different gene pools by using the frequency (*Fq*) of occurrence of genes among genomes, that is, the number of genomes in which a gene has a homolog. An *Fq* equal to 1 indicated that the gene had a homologous gene in every examined genome and could also be referred to as a strict core gene of the strains. The boundary of the accessory genome was determined to be 5%, indicating that one gene had a homologous gene in at least two genomes (45). A gene that has a homolog in more than 2 genomes but less than 44 genomes was classified into the character gene pool. Last, genes existing in less than or equal to two genomes could be categorized into the accessory genome. Genes that were not assigned to any orthogroups were unassigned genes, and species-specific genes did not have any homologous genes in other species, which could also be classified as unassigned genes. We made 10 combinations of genomes for each genome number, and the mean value of the 10 pangenome combinations was considered for estimating the pangenome curve. Using IBM SPSS Statistics 26, the pangenome curve was fitted by Heaps’ law as described by Tettelin et al.(46, 47). The equation of Heaps’ law can be described as “n = k*N^−α^,” where “n” represents the gene number of a given genome number (N), and k is a constant associated with the pangenome curve. If α is <1, the pattern of the pangenome would be considered “open.” In contrast, an α of >1 represents a closed pangenome.

### Genome annotation of S. maltophilia strains.

We used KAAS (https://www.genome.jp/tools/kaas/) to perform KEGG annotation, with a representative set selected “for prokaryotes.” The eggNOG 5.0 database was downloaded from the website (http://eggnog5.embl.de/download/emapperdb-5.0.0/), and the eggNOG V2 annotation was run locally.

### Phylogenetic tree construction for the 44 S. maltophilia strains.

The phylogenetic tree was generated by a built-in STAG algorithm in OrthoFinder. The Species Tree inference from all genes (STAG) method inferred a species tree by analyzing each multicopy gene family analyzed by OrthoFinder in turn (48), which could help to eliminate the inaccuracy of inference caused by the limited availability of single-copy orthogroups (i.e., orthogroups contain just one ortholog from each strain) (48). The phylogenetic tree was visualized by iTOL (49). Unless stated otherwise, software tools were used with their default settings.

### Gene category enrichment analysis.

Hypergeometric tests were performed based on gene annotation numbers to detect gene enrichment among the core genome, character genome, and accessory genome (*P* < 0.05). The hypergeometric tests were performed in R (version 4.0.2). To determine habitat adaptation features between two kinds of isolates, KEGG annotations were performed following the steps described in section “Genome annotation of S. maltophilia strains.” According to the gene annotation number of each category, enrichment analysis focusing on functional groupings of KEGG annotation was carried out using a *t* test (using IBM SPSS Statistics 26). Pairwise KEGG functional comparisons between clinical isolates and environmental isolates were inferred from two independent-sample *t* tests if gene annotation data were normally distributed (*P* < 0.05). Mann-Whitney U rank-sum tests were also performed if the annotation statistics in one category were not normally distributed.

### Mineral chemicals and culture media.

Mineral salt medium (MSM) and fluoranthene-MSM (FLA-MSM) cultures were prepared as described in our previous study (36). The concentration of FLA-MSM was set at 20 mg/liter, and glucose-MSM (Glu-MSM) culture was set at 5 g/liter.

### RNA extraction and real-time quantitative PCR.

The expression levels of PAH degradation-related genes were determined by RT-qPCR. The 16S rRNA gene was used as an internal reference to normalize the relative amount of target cDNA. Detailed information about the primers is given in Table S7 in the supplemental material. The total RNA of S. maltophilia W18 cultivated in FLA-MSM and Glu-MSM was extracted after 24 h of incubation using a bacterial RNA kit (Omega BioTek, USA). Total RNA was then reverse transcribed into cDNA using PrimeScript RT master mix (Perfect Real Time) (TaKaRa Biotech, Dalian). The reactions were performed on a Roche Diagnostics Lightcycler 480II fluorescence quantitative PCR system. A 20-μL PCR mixture containing 10-μL TB Green premix *Ex Taq* II(Tli RNaseH Plus) (2× concentration), 0.8 μL of each 10 μM primer pair, 0.2 μL of the cDNA template, and 8.2-μL double-distilled H_2_O (ddH_2_O) were prepared. The amplification protocol was performed according to the instruction manual of TB Green premix *Ex Taq* II (Tli RNaseH Plus) (TaKaRa Biotech). The qPCR analysis was conducted in triplicate reactions for all samples. The *Cp* value is the number of cycles required for the fluorescent signal the cross the threshold, which can be used to calculate relative genes expression level. The expression levels of the tested genes were determined by *Cp* values and calculated by the 2-delta delta *Cp* (2-ΔΔ*Cp*) method.

### Data availability.

The sequencing data were submitted to the NCBI database (https://submit.ncbi.nlm.nih.gov/subs/wgs/), and the GenBank accession number of the W18 strain is CP028358. All 44 completely sequenced S. maltophilia strains are available in the NCBI database.
