# Supplementary material for: Comparative Genomic Analysis of Stenotrophomonas maltophilia Strain W18 Reveals Its Adaptative Genomic Features for Degrading Polycyclic Aromatic Hydrocarbons
Source: Microbiol Spectr. 2021 Nov 24;9(3):e01420-21. doi: 10.1128/Spectrum.01420-21 (PMC8612148; doi:10.1128/Spectrum.01420-21)
Supplement: SUPPLEMENTAL FILE 9 — Supplemental material. Download SPECTRUM01420-21_Supp_9_seq12.pdf, PDF file, 0.1 MB [file spectrum01420-21_supp_9_seq12.pdf]

Supporting information reference

1. Niu B, Kolter R. 2017. Complete Genome Sequences of Seven Strains Composing a Model Bacterial Community of Maize Roots. Genome Announc 5.https://doi.org/10.1128/genomeA.00997-17
2. Gröschel MI, Meehan CJ, Barilar I. 2020. The phylogenetic landscape and nosocomial spread of the multidrug-resistant opportunist Stenotrophomonas maltophilia. Nat Commun 11.https://doi.org/10.1038/s41467-020-15123-0
3. Pallavi Subhraveti, Quang Ong, Ingrid Keseler, Anamika Kothari, Ron Caspi, Karp PD. Summary of Stenotrophomonas maltophilia, Strain JV3, version 24.5. https://biocyc.org/SMAL868597/organism-summary?object=SMAL868597. Accessed
4. Lira F, Hernandez A, Belda E, Sanchez MB, Moya A, Silva FJ, Martinez JL. 2012. Whole-genome sequence of Stenotrophomonas maltophilia D457, a clinical isolate and a model strain. J Bacteriol 194:3563-4.https://doi.org/10.1128/JB.00602-12
5. Subhraveti P, Midford P, Keseler I, Kothari A, Caspi R, Karp PD. Summary of Stenotrophomonas maltophilia, Strain NCTC 10259, version 24.5. https://biocyc.org/organism-summary?object=GCF_900636905. Accessed
6. Pak TR, Altman DR, Attie O, Sebra R, Hamula CL, Lewis M, Deikus G, Newman LC, Fang G, Hand J, Patel G, Wallach F, Schadt EE, Huprikar S, van Bakel H, Kasarskis A, Bashir A. 2015. Whole-genome sequencing identifies emergence of a quinolone resistance mutation in a case of Stenotrophomonas maltophilia bacteremia. Antimicrob Agents Chemother 59:7117-7120.https://doi.org/10.1128/AAC.01723-15
7. Elufisan TO, Lozano L, Bustos P, Rodríguez-Luna IC, Sánchez-Varela A, Oyedara OO, Villalobos-López MÁ, Guo X. 2019. Complete Genome Sequence of Stenotrophomonas maltophilia Strain SVIA2, Isolated from Crude Oil-Contaminated Soil in Tabasco, Mexico. Microbiol Resour Announc 8.https://doi.org/10.1128/MRA.00529-19.
8. Permala R.R, Glady-Croue J, Watkin E.L.J, J.P R, J.P C. 2018. Complete genome sequence of Stenotrophomonas maltophilia AB550, an environmental solar radiation- and multidrug-resistant strain isolated in Western Australia. Microbiol Resour Announc 7.https://doi.org/10.1128/MRA.00914-18.
9. Hamidian M, Lazenby J, To J, Hartstein R, Soares J, McNamara S, Whitchurch CB. 2020. Complete Genome Sequence of Stenotrophomonas maltophilia Strain CF13, Recovered from Sputum from an Australian Cystic Fibrosis Patient. Microbiol Resour Announc 9.https://doi.org/10.1128/MRA.00628-20
10. Kang WN, Fu KY, Guo WC, Li GQ. 2020. Complete Genome Sequence of Stenotrophomonas maltophilia Strain CPBW01, Isolated from the Wings of the Colorado Potato Beetle in Xinjiang, China. Microbiol Resour Announc 9.https://doi.org/10.1128/MRA.00460-20
11. Sassera D, Leardini I, Villa L, Comandatore F, Carta C, Almeida A, do Céu Sousa M, Gaiarsa S, Marone P, Pozio E, S.M C. 2013. Draft genome sequence of Stenotrophomonas maltophilia strain EPM1, found in association with a culture of the human parasite Giardia duodenalis. Genome Announc 1.https://doi.org/10.1128/genomeA.00182-13
12. Brooke JS. 2012. Stenotrophomonas maltophilia: an Emerging Global Opportunistic Pathogen. Clin Microbiol Rev 25.https://doi.org/10.1128/CMR.00019-11
13. Xiong WL, C Y, W.L P, Z.X D, S.J L, R.B L. 2020. Characterization of an 17β-estradiol-

degrading bacterium Stenotrophomonas maltophilia SJTL3 tolerant to adverse environmental factors. Appl Microbiol Biotechnol 104:15.https://doi.org/10.1007/s00253-019-10281-8

14. Dong H, Rui JP, Sun JN, Li XZ, Mao XZ. 2017. Complete genome sequencing and diversity analysis of lipolytic enzymes in Stenotrophomonas maltophilia OUC_Est10 Acta Microbiologica Sinica 57:1716-1721.https://doi.org/10.13343/j.cnki.wsxb.20170220

15. Subhraveti P, Midford P, Keseler I, Kothari A, Caspi R, Karp PD. Summary of Stenotrophomonas maltophilia, Strain NCTC13014, version 24.5. https://biocyc.org/organism-summary?object=GCF_900636655. Accessed

16. Lucas S CA, Lapidus A, Glavina del Rio T, Dalin E, Tice H, Pitluck S, Chain P, Malfatti S, Shin M, Vergez L, Lang D, Schmutz J, Larimer F, Land M, Hauser L, Kyrpides N, Mikhailova N, Taghavi S, Monchy S, Newman L, Vangronsveld J, van der Lelie D, Richardson P. Proteomes - Stenotrophomonas maltophilia (strain R551-3). https://www.uniprot.org/proteomes/UP000001867. Accessed

17. Davenport KW, Daligault HE, Minogue TD, Broomall SM, Bruce DC, Chain PS, Coyne SR, Gibbons HS, Jaissle J, Li PE, Rosenzweig CN, Scholz MB, Johnson SL. 2014. Complete Genome Sequence of Stenotrophomonas maltophilia Type Strain 810-2 (ATCC 13637). Genome Announc 2.https://doi.org/10.1128/genomeA.00974-14

18. Guan JJ, Wang HQ, Zhu Y, Xu J. 2019. Whole-genome Sequencing of S.maltophilia and Degradation of Fluoranthene. Genomics and Applied Biology 38:3037-3045.https://doi.org/10.13417/j.gab.038.003037

19. Xiong W, Yin C, Wang Y, Lin S, Deng Z, Liang R. 2020. Characterization of an efficient estrogen-degrading bacterium Stenotrophomonas maltophilia SJTH1 in saline-, alkaline-, heavy metal-contained environments or solid soil and identification of four 17beta-estradiol-oxidizing dehydrogenases. J Hazard Mater 385:121616.https://doi.org/10.1016/j.jhazmat.2019.121616

20. Peng T, Kan J, Hu J, Hu Z. 2020. Genes and novel sRNAs involved in PAHs degradation in marine bacteria Rhodococcus sp. P14 revealed by the genome and transcriptome analysis. 3 Biotech 10:140.https://doi.org/10.1007/s13205-020-2133-6

21. Hasson MS, Schlichting I, Moulai J, Taylor K, Barrett W, Kenyon GL, Babbitt PC, Gerlt JA, Petsko GA, Ringe D. 1998. Evolution of an enzyme active site: the structure of a new crystal form of muconate lactonizing enzyme compared with mandelate racemase and enolase. Proc Natl Acad Sci U S A 95:10396-401.https://10.1073/pnas.95.18.10396

22. Dasari S, Ganjayi MS, Yellanurkonda P, Basha S, Meriga B. 2018. Role of glutathione S-transferases in detoxification of a polycyclic aromatic hydrocarbon, methylcholanthrene. Chem Biol Interact 294:81-90.https://doi.org/10.1016/j.cbi.2018.08.023

23. Holmquist M. 2000. Alpha/Beta-Hydrolase Fold Enzymes: Structures, Functions and Mechanisms. Curr Protein Pept Sci 1:209-235.https://doi.org/10.2174/1389203003381405

24. Li XL, He YC, Zhang LB, Xu ZY, Ben HX, Gaffrey MJ, Yang YF, Yang SH, Yuan JS, Qian WJ, Yang B. 2019. Discovery of potential pathways for biological conversion of poplar wood into lipids by co-fermentation of Rhodococci strains. Biotechnology for Biofuels 12.https://10.1186/s13068-019-1395-x

25. Poroca DR, Pelis RM, Chappe VM. 2017. CIC Channels and Transporters: Structure, Physiological Functions, and Implications in Human Chloride Channelopathies. Frontiers In Pharmacology 8.https://10.3389/fphar.2017.00151

26. Kim HK, Harshey RM. 2016. A Diguanylate Cyclase Acts as a Cell Division Inhibitor in a Two-

Step Response to Reductive and Envelope Stresses. mBio 7.https://10.1128/mBio.00822-16
27. Torres-Farrada G, Manzano-Leon AM, Rineau F, Ramos Leal M, Thijs S, Jambon I, Put J, Czech J, Guerra Rivera G, Carleer R, Vangronsveld J. 2019. Biodegradation of polycyclic aromatic hydrocarbons by native Ganoderma sp. strains: identification of metabolites and proposed degradation pathways. Appl Microbiol Biotechnol 103:7203-7215.https://10.1007/s00253-019-09968-9
28. Stanier RY, Gunsalus IC, Gunsalus CF. 1953. The enzymatic conversion of mandelic acid to benzoic acid. II. Properties of the particulate fractions. J Bacteriol 66:543-7.http://10.1128/jb.66.5.543-547.1953
29. Berleman JE, Bauer CE. 2005. Involvement of a Che-like signal transduction cascade in regulating cyst cell development in Rhodospirillum centenum. Mol Microbiol 56:1457-66.https://10.1111/j.1365-2958.2005.04646.x
30. Luu RA, Kootstra JD, Nesteryuk V, Brunton CN, Parales JV, Ditty JL, Parales RE. 2015. Integration of chemotaxis, transport and catabolism in Pseudomonas putida and identification of the aromatic acid chemoreceptor PcaY. Mol Microbiol 96:134-47.https://10.1111/mmi.12929
31. Tropel D, van der Meer JR. 2004. Bacterial transcriptional regulators for degradation pathways of aromatic compounds. Microbiol Mol Biol Rev 68:474-500, table of contents.https://10.1128/MMBR.68.3.474-500.2004
32. Pan JL, Bardwell JC. 2006. The origami of thioredoxin-like folds. Protein Sci 15:2217-27.https://10.1110/ps.062268106
